# Low-Cost, Distributed Environmental Monitors for Factory Worker Health

**DOI:** 10.3390/s18051411

**Published:** 2018-05-03

**Authors:** Geb W. Thomas, Sinan Sousan, Marcus Tatum, Xiaoxing Liu, Christopher Zuidema, Mitchell Fitzpatrick, Kirsten A. Koehler, Thomas M. Peters

**Affiliations:** 1 Department of Mechanical and Industrial Engineering, The University of Iowa, Iowa City, IA 52242, USA; marcus-tatum@uiowa.edu (M.T.); xiaoxing-liu@uiowa.edu (X.L.); mitchell-fitzpatrick@uiowa.edu (M.F.); 2 Department of Occupational and Environmental Health, The University of Iowa, Iowa City, IA 52242, USA; sinan-jameel@uiowa.edu (S.S.); thomas-m-peters@uiowa.edu (T.M.P.); 3 Department of Mathematics and Computer Science, Adelphi University, New York, NY 11530, USA; 4 Department of Environmental Health & Engineering, Johns Hopkins University, Baltimore, MD 21205, USA; czuidema@jhu.edu (C.Z.); kkoehle1@jhu.edu (K.A.K.)

**Keywords:** wireless sensor network, occupational health and safety, sensor arrays, particle sensors, carbon monoxide gas sensors, oxidizing gas sensors, noise sensors, occupational medicine, aerosol exposure, personal exposure

## Abstract

An integrated network of environmental monitors was developed to continuously measure several airborne hazards in a manufacturing facility. The monitors integrated low-cost sensors to measure particulate matter, carbon monoxide, ozone and nitrogen dioxide, noise, temperature and humidity. The monitors were developed and tested in situ for three months in several overlapping deployments, before a full cohort of 40 was deployed in a heavy vehicle manufacturing facility for a year of data collection. The monitors collect data from each sensor and report them to a central database every 5 min. The work includes an experimental validation of the particle, gas and noise monitors. The *R*^2^ for the particle sensor ranges between 0.98 and 0.99 for particle mass densities up to 300 μg/m^3^. The *R*^2^ for the carbon monoxide sensor is 0.99 for concentrations up to 15 ppm. The R^2^ for the oxidizing gas sensor is 0.98 over the sensitive range from 20 to 180 ppb. The noise monitor is precise within 1% between 65 and 95 dBA. This work demonstrates the capability of distributed monitoring as a means to examine exposure variability in both space and time, building an important preliminary step towards a new approach for workplace hazard monitoring.

## 1. Introduction

Exposure to air pollutants and physical agents is the cause for various health risks in many working environments. Environmental hazards in the workplace include particulate matter (PM), particles that can penetrate the alveolar regions of the lungs, oxidative gases, carbon monoxide, extreme temperatures, and noise. Aerosol exposure is associated with increased mortality rates [1], cardiopulmonary diseases [2,3,4,5,6,7], and neurological effects [8,9]. Ozone (O_3_) and nitrogen oxides (NO and NO_2_) are associated with an increased risk of pulmonary function [10], eyes, nose, and throat irritation [11], and DNA damage [12]. Carbon monoxide poisoning causes mild to severe morbidity, including headaches, chest pain, and cardiac or respiratory arrest [13]. Sulfur dioxide (SO_2_) exposure is associated with various respiratory effects [11]. Excessive noise may lead to irreversible hearing loss, elevated blood pressure, sleep deprivation, and stress [14].

Industrial hygienists use personal sampling as the primary means to ensure that a working population is protected from such hazards. For aerosols and gases, personal sampling of breathing zone air typically results in a time-weighted average measure of personal exposure that can be compared to an occupational exposure limit. The permissible exposure limits for aerosols and gases as set by the Occupational Safety and Health Administration (OSHA) is an 8-h, time-weighted average (TWA) [15]. However, for acute hazard measurements, shorter periods (5-min or 15-min) are necessary to capture personal worker exposure [16]. These personal sampling methods are often limited to very few workers and very few sample intervals because of: (1) costs; (2) a log-normal distribution of exposures that increases the likelihood of non-compliance as more exposures are measured; and (3) a regulatory framework not requiring sufficient samples to capture exposure variability [17]. In the nickel producing industry, for example, Tornero-Velez et al. [18] found that the median number of personal samples taken in annual surveys was 1, and 95% had 10 or fewer annually, a situation that is getting worse [19]. Thus, the industrial hygienist routinely makes decisions about worker protection with relatively little information, rendering such decisions unreliable. Rezagholi and Mathiassen [20] stressed that more cost-effective, but less accurate and precise, methods will increase sample size and improve exposure assessment. Understanding variations in exposure is critical to identifying the determinants of exposure and devising effective risk mitigation strategies.

Direct-reading instruments, sensors that provide near real-time output of hazard concentration or intensity, can be applied to assess exposure variability through task (temporal) monitoring or hazard mapping (depiction of concentrations measured spatially as contours on a map). However, direct-reading instruments have found limited use in industrial hygiene practice because they have traditionally been relatively expensive (>$4500 each). The approach described in this paper, one that relies on a network of relatively low-cost sensors, is a cost-effective exposure assessment framework that has been recognized by other researchers [21,22,23,24,25], and rapidly assesses workplace aerosol hazards with unprecedented spatiotemporal resolution for the large size of the measurement area.

This system advances the work of other researchers (e.g., [21,22,23,24,25,26,27,28,29,30,31,32,33,34,35,36]) in several ways. First, rather than demonstrating a breadboard prototype, we have developed and deployed 40 integrated devices capable of long-term performance in a factory environment. The architecture emphasizes transmitting data to a central repository, a practical approach in a controlled network environment. In addition to the particulate matter and temperature and humidity sensors used in previous work, we have integrated a customized noise level sensor and two scientific-quality gas sensors, sensors capable of detecting low levels of ozone and carbon monoxide. Each of the sensors was tested after integration on the customized circuit board, in the case of the gas sensors, and as part of the integrated module, in the case of the particulate and noise sensors.

In this paper we describe important design considerations for the development of the monitors, explain our procedure to check each of the sensors’ response against high-quality direct-reading instruments in the lab before deployment, and demonstrate the utility of a sensor network by providing some preliminary examples of field data that can be produced. The calibration procedures, details of sensor performance, and complete analysis of the data produced from the sensors are reported elsewhere e.g., [37,38,39,40] or are being prepared for publication.

## 2. Materials and Methods

The objective of the monitor design was to support a sensor network of 40 units operating in a large manufacturing plant, with each sensor consisting of parts costing a few hundred dollars. The final design includes sensors for airborne particles, carbon monoxide, oxidizing gases, temperature, humidity, and noise integrated within a 20 cm × 10 cm × 7.5 cm sealed plastic case. These integrated devices are powered with a 5 V USB power adaptor and communicate wirelessly over the factory’s existing wireless infrastructure using HTTP calls through the OpenWRT operating system. The devices are programmed to check the server and network status periodically and are able to recover from a network or power failure. Sensor values recorded by the monitors are stored in a central database. This report focuses on the design of the monitors and the distributed network.

### 2.1. Monitor Design Requirements

In integrating the sensors into the monitors, it was important to consider a variety of factors that would affect their long-term use. For example, the monitors needed to be inconspicuous and positioned to avoid being inadvertently hit or damaged on the busy factory floor. After working through several options with factory personnel, we designed the monitors to fit between the webs of the I-beam columns that supported the factory roof. Another factor affecting the monitor design was that the circuitry in the monitors needed to be protected from dust and debris. To some extent, this requirement conflicted with the need for the dust sensors to have direct access to the environmental air, which meant including an inlet and outlet to channel air through the device. The gas sensors also needed to be exposed to the environment, but in a manner that avoided allowing large particles to accumulate on their filters. The monitor’s protected position inside the I-beam webbing potentially baffled sounds from the environment, so the microphone needed to extend far enough away from the I-beam that the column would not muffle sound from sources on either side. Finally, the monitors required the ability to communicate with the wireless network to store all their data in a central server as well as the capacity to support an on-board flash drive backup.

### 2.2. Selection of Sensors

The sensors were selected to monitor and assess the most likely hazards for workers in a heavy manufacturing plant involving metal cutting, grinding, and welding. Welding activities generate metal aerosols and gas pollutants such as carbon monoxide, nitrogen oxides, sulfur dioxide, and ozone. At the time the study was conducted, and after careful review of the available low-cost aerosol and gas sensors, our team selected a dust sensor (Sharp, Model DN7C3CA006, Osaka, Japan) capable of detecting particulate matter with an average diameter of less than 2.5 microns (PM2.5), a 4-electrode carbon monoxide sensor (Alphasense, Model CO-B4 with sensor board 000-01SB-02, Essex, UK), a 4-electrode oxidizing gas sensor (Alphasense, model OX-B431 with sensor board 000-01SB-02, Essex, UK), a temperature and humidity sensor (Adafruit, model AM2302, New York, NY, USA), and a custom-built noise level sensor built from a Teensy 3.2 Microprocessor with an omnidirectional condenser microphone (CUI Inc., CMA-4544PF-W, Tualatin, WA, USA).

Sharp produces two configurations for its dust sensor: the GP series and the DN series. The GP series (e.g., model GP2Y10101AU0F), commonly used in previous research, includes just the optical sensing arrangement with passive airflow. The DN series adds a fan on the outlet and a virtual impactor on the input that only permits particles smaller than 2.5 microns to enter the sensing zone. Initially, the Sharp DN sensor was selected after comparison with several other low-cost sensors [37]. This report describes the performance of the DN sensor. The original experiments with the DN series sensors are described here because the sensor is likely to be useful to others working in slightly less contaminated environments. To date, these experiments have not been reported elsewhere.

The carbon monoxide and oxidizing gas sensors were selected based on their sensitivity and compactness. The oxidizing gas sensor measures the sum of O_3_ and NO_2_. At the time the oxidizing gas sensors were selected, the main commercial providers of electrochemical gas sensors only offered one sensor that responded to both ozone and nitrogen dioxide [41]. Subsequent to the construction of the monitors in this network, the manufacturer developed a sensor specific to nitrogen dioxide and proposed pairing an oxidizing gas sensor with a nitrogen dioxide sensor to obtain more accurate measurements of both gases [42]. The temperature and humidity sensors were included for their potential to improve gas sensor measurements.

The noise sensor was purpose-built from components because we were unable to identify an appropriately packaged noise sensor that could be integrated with the rest of the equipment [38].

### 2.3. Hardware Integration

**Figure 1:**
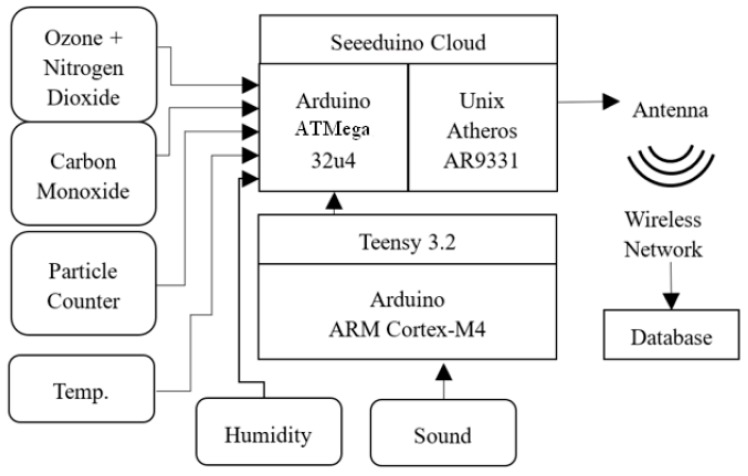
A block diagram of the sensor and microprocessor architecture.

The system (Figure 1) was designed to provide sensor reports from each monitor once every five minutes.

**Figure 2:**
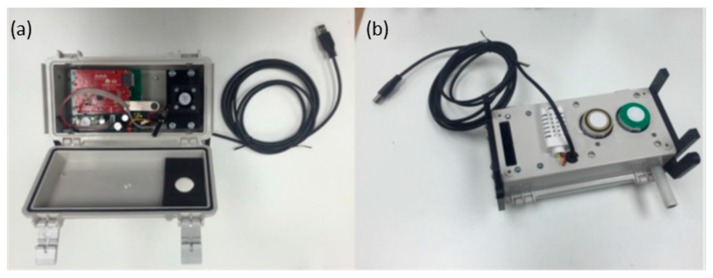
The monitor is encased in a plastic box. (**a**) The open top view reveals the location of the main electronic components and the body of the Sharp DN particle sensor and fan assembly. (**b**) The bottom view reveals the position of the sensors and the black mounting brackets on either end.

Every two seconds, the microprocessor (ATmega32u4) in each monitor collects the analog and digital input data from the four sensors, as well as the serial report from the Teensy microprocessor analyzing the microphone data and passes these data to a WiFi module (Dragino WiFi IoT module HE), which is programmed with a lightweight Unix variant, OpenWrt. The Unix system averages the data over a five-minute interval and transmits the averages to a central server. The noise monitoring is too computationally intense for the Seeeduino microprocessor; instead, the fast Fourier transform of the signal is processed on the Teensy 3.2′s 32-bit ARM Cortex-M4 72 MHz processor. A printed circuit was designed and manufactured to incorporate all the devices and microcontrollers into a single package that can easily fit into a compact enclosure (Figure 2).

Our circuit board included the option of amplifying the carbon monoxide and oxidizing gas sensors, signals both initially refined with the manufacture’s sensor board. This amplification trades sensor range for precision as the two signals (designated as OP1 and OP2 by the board manufacturer) are scaled to the 5 V maximum input on the microprocessor’s analog-to-digital convertor. After considering past measurements in the factory, the gains were set to 1:1 for the carbon monoxide sensors and 1:5 for the oxidizing gas sensors. Consequently, sensor results larger than the expected range were measured at the maximum sensor value.

**Figure 3:**
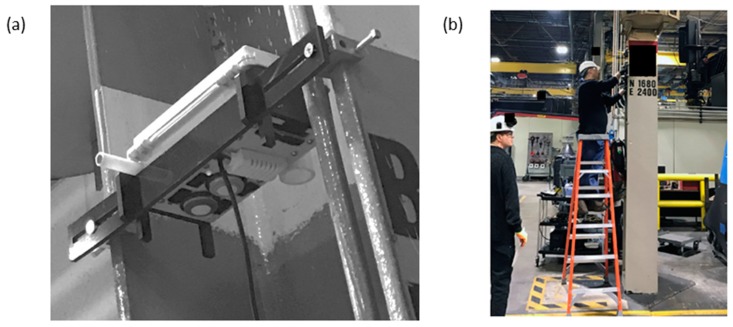
(**a**) Monitor shown mounted in the factory, clamped between two flanges of a support I-beam, positioned about 2.5 m above the ground. The microphone extends out from the beam. The other sensors are visible on the underside of the monitor. The I-beams were spaced 30–40 m from one another. (**b**) Monitor deployment by the authors (on top of a ladder) in the working environment.

The central microprocessor and sensors rely on a 5 V power supply and typically consume 1 W of power during operation. The monitors were designed to be mounted in the web of an I-beam approximately 2.5 m above the ground (Figure 3) and covered a workspace that measured approximately 252 m × 292 m with a ceiling approximately 8.5 m high. A bracket-and-hook system was developed to secure the monitors to the brackets at a fixed location and orientation, while simultaneously allowing for them to be retrieved with minimal effort. To prevent safety concerns, the monitors were designed to allow for a pole to be able to place and retrieve the monitors, negating the need for a ladder or stepping stool.

### 2.4. Sensor Performance

The particle and gas sensors were evaluated in six experiments using the exposure chamber described below.

**Figure 4:**
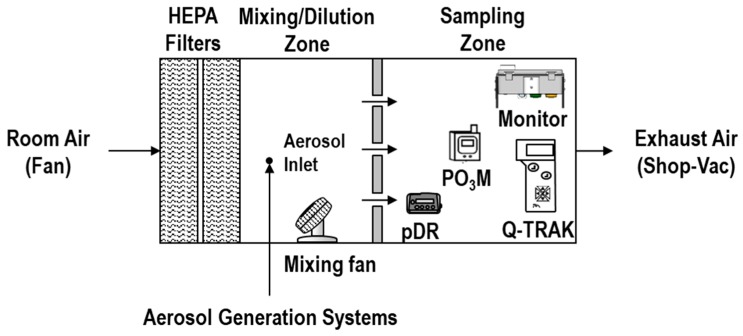
Experimental set up used to determine the performance of the monitors.

The chamber used in experiment 1 consists of a mixing zone (0.64 m × 0.64 m × 0.66 m) and a sampling zone (0.53 m × 0.64 m × 0.66 m). The experimental setup is shown in Figure 4. The mixing zone and sampling zone were divided by a perforated plate positioned in the middle. The perforated plate provided adequate mixing inside the sampling zone to avoid dead zones. The low-cost monitors and the reference instruments were positioned inside the sampling zone of the chamber. A total of 30 monitors were evaluated at a time. Six stacks of five monitors each were positioned in two identical rows. The purpose of these laboratory experiments was to show sensor response compared to the reference instruments. For the particulate sensors, we show response in mV compared to concentration according to a reference instrument. For the gas sensors, we use previously-derived calibration curves to convert sensor signal to concentration for comparison with a reference instrument [39].

#### 2.4.1. Particulate Sensors

The Sharp DN particulate matter sensor response was evaluated with salt, a common laboratory test aerosol [37]. Salt particles were generated with a vibrating nebulizer (Aerogen, Aeroneb Solo System, Galway, Ireland) and a 0.9% salt solution (Fisher Scientific, *w*/*v*, #7210, Hampton, NH, USA). The aerosol was diluted with clean air from two HEPA filters (0.25 m^3^/min) positioned at the entrance of the sampling zone. Salt size distribution and median diameter were not measured for this experiment. Measurements of the particle mass concentrations were compared to a personal DataRAM 1500 Aerosol Monitor (Thermo Fisher Scientific., pDR, Shoreview, MN, USA). Prior to our experiment, the pDR was sent to the manufacturer for calibration, and was operated with an inlet cyclone (cut-off diameter of 10 mm). Sousan et al. [39] evaluated the pDR compared to filter-corrected, high-cost reference instruments for the same salt solution used and found that the pDR measurements were linear and had a correlation of 0.99, a slope of 1.1, and a bias of just 6% compared to the reference instruments. Clean air (pDR concentration = 0.01 µg/m^3^) was measured inside the chamber for five minutes before generating salt aerosols. The concentration range for the salt aerosol inside the chamber varied from 0 to 300 μg/m^3^, concentration levels consistent with earlier measurements in the factory environment. The mean temperature and relative humidity inside the chamber was 24 °C (standard deviation (SD) = 0.4 °C) and 21% (SD = 1.7%), respectively. We therefore expect that temperature and relative humidity effects were negligible. Steady-state concentrations were maintained at six levels for five minutes in order to eliminate response time differences between the Sharp DN sensors and the pDR. The Sharp DN sensors recorded a measurement every two seconds; the pDR recorded a measurement every second. Five-minute averages were calculated for every sensor at each steady state level, comparing slopes, intercepts, and R-values. Sousan et al. [37] evaluated the Sharp DN sensors with different aerosol types and found that each sensor needed to be calibrated once purchased from the manufacturer. Also, the Sharp DN sensor correlation was similar with a linear response for different aerosol types; however, the magnitude (in voltage) was higher for reflective aerosols, such as salt, and lower for darker absorbing aerosols, such as welding and diesel fumes. Therefore, the authors concluded that the Sharp DN sensors should be calibrated on-site with a reference instrument.

#### 2.4.2. Carbon Monoxide Sensors

To measure sensor response of the carbon monoxide sensors, a MAP-Pro Hand Torch Cylinder (BernzOmatic Company, Newark, NJ, USA) was used to generate carbon monoxide gas inside the chamber. Measurements from the CO sensors were compared to a Q-Trak Plus 8552 (TSI Inc., Shoreview, MN, USA) reference instrument. The Q-Trak is an electrochemical sensor and was calibrated with zero air (GASCO 103L-1, Oldsmar, FL, USA) and a 10-ppm calibration gas tank (GASCO 103L-375A-10, Oldsmar, FL, USA) before the experiment. The torch was operated for 150 s and measurements were collected as the CO slowly dissipated in the still air of the chamber. The CO sensor measurements were converted from mV to ppm using slope (0.358 ppm/V) and intercept (0.217 mV) values from earlier laboratory experiments [40]. In that study, Afshar-Mohajer et al. exposed three CO sensors at concentration levels of 2, 5, 10, 12, 15, 25, 30 and 50 ppm, and compared the results with a GrayWolf Advanced Pro (GrayWolf Sensing Solutions LLC, Shelton, CT, USA) reference instrument. The authors of that study then derived a linear regression model with high correlation (*R*^2^ = 0.998) between the CO sensor and the GrayWolf Advanced Pro measurements.

#### 2.4.3. Oxidizing Gas Sensors

To measure sensor response of the oxidizing gas sensors, an AQUA-6 ozone generator (A2Z Ozone, Inc., Louisville, KY, USA) was used to generate ozone gas inside the chamber. Sensor measurements were compared to those observed with a Personal Ozone Monitor (2B Technologies Inc., PO3M, Boulder, CO, USA) reference instrument. The ozone generator was operated for five seconds and measurements were collected as the generated ozone dissipated. The conversion from mV to ppb was based on slope and intercept values from earlier laboratory experiments in which three sensors were exposed to ozone at concentration levels of 25, 50, 75 and 100 ppb and separate experiments where sensors were exposed to nitrogen dioxide concentrations of 0.2, 0.5, 1 and 1.5 ppm [40]. For this ozone decay experiment, we applied the sensors’ relationship with O_3_. Since the selection of the oxidizing gas sensors for this network, the manufacturer has proposed pairing the oxidizing gas sensor with a nitrogen dioxide sensor to improve the accuracy of measurements of both gases [42]. Evaluation of the paired oxidizing gas and nitrogen dioxide sensors to measure ozone and nitrogen dioxide in mixture will be evaluated in future studies.

#### 2.4.4. Noise Sensors

The noise sensors were tested in a laboratory protocol based on a NIOSH evaluation of smartphone noise measurement applications [43]. Measurements from the low-cost noise sensors were compared to a reference sound level meter (NTi Audio, SLM, Schaan, Liechtenstein). Pink noise was generated using a TalkBox (NTI Audio, Schaan, Liechtenstein) and an amplifier (Fender Musical Instruments Corp., Scottsdale, AZ, USA). Side-by-side measurements were conducted for each low-cost noise sensor and the reference instrument. The noise sensors were positioned 15 cm away from the TalkBox and amplifier. Pink noise was generated between 65 and 95 dB in 5-dB increments, and for 30 s at each sound level. Average slope, intercept, R-value, bias and precision were calculated between the low-cost noise sensors and the reference instrument, with an acceptance criterion of ±2 dB [44].

#### 2.4.5. Gas Sensor Bias and Precision

For the gas sensors, bias and precision experiments were conducted outside the chamber using a shroud and a reference gas for one minute. NIOSH evaluation for gas sensors requires that the test be performed with a known gas concentration and that the measurements be conducted at steady state concentrations due to response time differences between instruments. Therefore, due to the unavailability of ozone calibration gas, we were only able to deliver CO and NO_2_ gas at stable conditions to the sensors, using calibration gases. For the CO sensors, a 10-ppm carbon monoxide span calibration gas (Gasco, part number 103L-375-10, Oldsmar, FL, USA) was used. For the oxidizing gas sensors, a 2-ppm nitrogen dioxide span calibration gas (Gasco, part number 58L-112-2, Oldsmar, FL, USA) was used because an O_3_ reference gas was not available, and we were unable to generate known steady-state O_3_ concentrations. To convert sensor signal to NO_2_ concentration for this experiment, we applied the sensors’ relationship with NO_2_. Measurements were collected from each low-cost sensor for one minute. The bias (*B*) was calculated as [45]:

(1) $$B=\frac{1}{n}\sum \frac{y_{i}-x_{i}}{x_{i}}$$

where *y* is the calculated mass concentration for the sensor, *x* is the reference gas concentration, and *n* is the number of data pairs measured in one minute. Bias ranges, minimum, and maximum values were calculated for each sensor.

Precision represents the variation among sensors and was calculated as the measure of the coefficient of variation (*CV*) of the calculated mass concentrations. Based on the one-minute data, the *CV* was calculated as [46]:

(2) $$CV=\;\frac{\sigma }{\text{µ}}$$

where *σ* is the standard deviation and *µ* is the mean of the mass concentration measurements from the 30 replicate sensors of the same type.

### 2.5. Field Deployment

The network was installed within the fabrication area of a manufacturing facility that produces heavy vehicles for construction and forestry. Work processes in the factory include welding, cutting, machining, grinding and abrasive blasting. The monitors in our network were deployed in the facility in a spatially optimized pattern to capture maximum spatial variability and reduce monitor redundancy [47]. While the network was deployed in stages, the complete network of 40 monitors was deployed for seven months. Here we provide examples of preliminary field measurements to display the type of data the sensor network can provide. Of particular importance are hazard maps, which display measured hazards throughout a facility or geographic area through maps [48,49,50,51]. Presentation and analysis of the long-term temporospatial variability of hazards in the facility, sensor accuracy, and sensor precision are forthcoming [52]. Calibration curves developed in prior work were used to translate sensor signal in the field to concentration or sound pressure level [38,40,53].

## 3. Results and Discussion

### 3.1. Particulate Sensors

**Figure 5:**
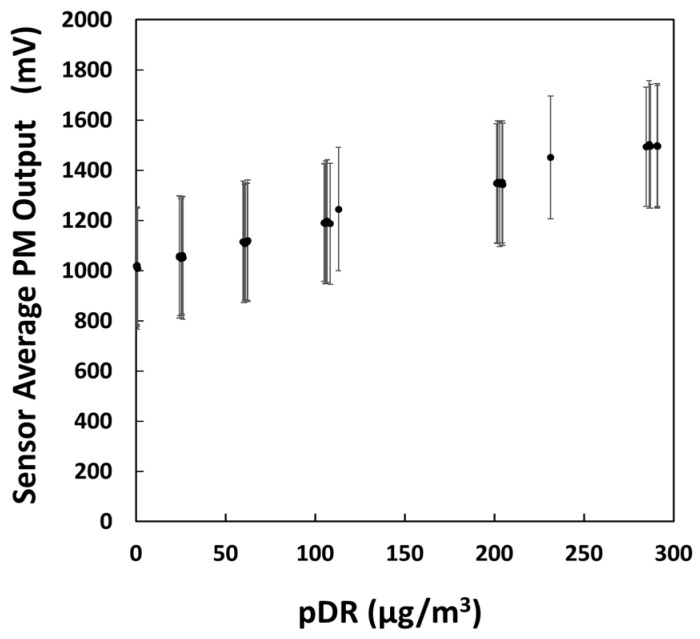
Sharp DN mV outputs for each monitor (*n* = 30) relative to the particulate mass reported by the reference aerosol monitor for varying levels of salt concentrations.

A scatterplot of the average Sharp DN outputs relative to the pDR sensor, as observed during the salt experiment, is shown in Figure 5. Each point in the scatterplot represents the average value reported by the 30 sensors. The *y*-axis error bars represent the standard deviation between sensors.

The R^2^ of the PM sensors ranged between 0.98 and 0.99. The fit slopes varied between 1.0 and 2.4 mV/µg/m^3^. The fit intercepts varied between 662 and 1778 mV.

Wide standard deviations between the monitors was insufficient to discriminate between practically important differences in particle mass concentration. However, by individually calibrating each sensor to the reference instrument, it was possible to adequately discriminate among substantially different levels of particulate mass density [37].

### 3.2. Carbon Monoxide Sensors

**Figure 6:**
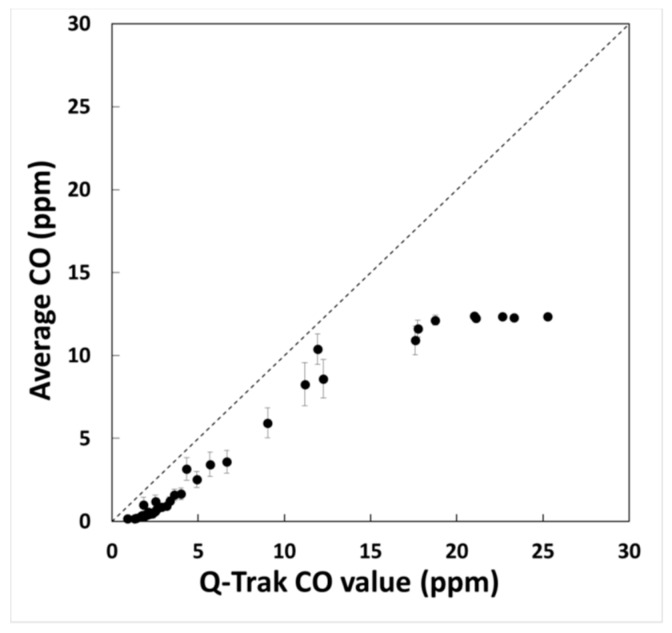
Decay test results of the average CO concentration for all sensors relative to the Q-Trak Plus 8552 reference measurements. The y-axis error bars represent one standard deviation.

Figure 6 illustrates the average concentration of 30 sensors and their standard deviations relative to the Q-Trak Plus 8552 reference instrument during the CO decay experiment. The leveling off indicates that, for the selected gain on the monitor board, the low-cost CO sensors were saturated for concentrations above 15 ppm. Therefore, we performed linear regression comparing measurements from the low-cost CO sensors and the Q-Trak Plus 8552 for concentrations below 15 ppm, and the average slope, intercept, and *R*^2^ were equal to 0.85, −1.3 ppm, and 0.99, respectively. For the span gas experiment, the bias values varied from 4% to 27%, and the precision for all monitors was 5%.

The CO sensors in the monitors are limited to concentration values below 15 ppm. This is low relative to the OSHA recommended time-weighted average value of 50 ppm [54], but sufficient for the expected levels of carbon monoxide in the setting we were investigating. The correlation was high among the CO sensors, suggesting that the average calibration constants derived for all sensors were adequate [40]. The bias variability may be related to the fact that we used an average slope and intercept for all the sensors, where in reality, each sensor has its unique slope and intercept.

### 3.3. Oxidizing Gas Sensors

**Figure 7:**
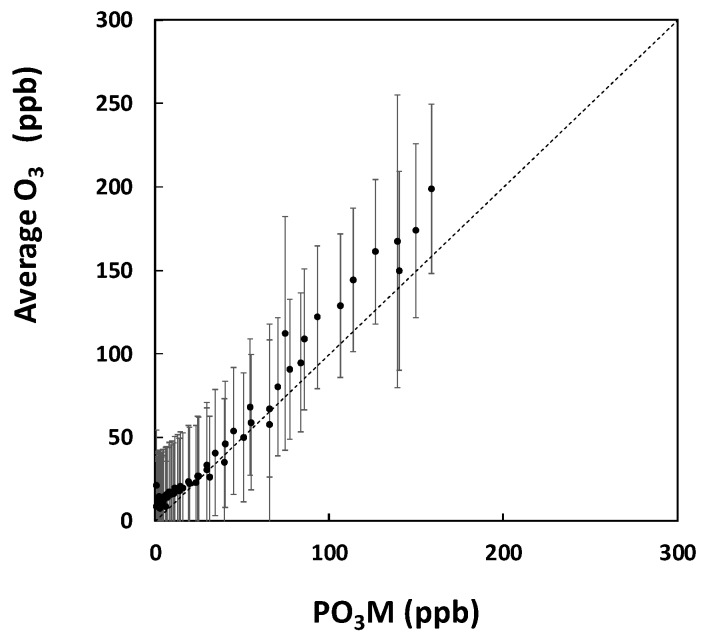
Decay test results for the average O_3_ concentration for all sensors relative to the reference measurements from the Personal Ozone Monitor (PO_3_M). The error bars represent one standard deviation.

Figure 7 presents the average concentrations and standard deviations of the low-cost sensors relative to the Personal Ozone Monitor during the ozone decay experiment. Average concentration levels and the standard deviation (*y*-axis error bars) were calculated for all sensors. The average slope, intercept, and *R*^2^ were 1.1, 5.4 ppm and 0.98, respectively. The decay experiment was exponential, so there was more variability at high concentrations than lower concentrations. The variability in sensor response reflects the variability in ozone concentration during the measurement period. In addition, each sensor has a unique response compared to the average slope applied to the sensors for this work. For the NO_2_ span gas experiment, the bias values varied from −39% to −11%, and the precision for all monitors was 11%.

The sensors were highly correlated with the reference instrument throughout the measurement range of interest of 20–180 ppb. The bias variability and lower precision of these sensors compared to the CO sensors is probably a consequence of using one calibration equation for all sensors when in reality there is substantial variability in response among the oxidative gas sensors.

### 3.4. Noise Sensors

**Figure 8:**
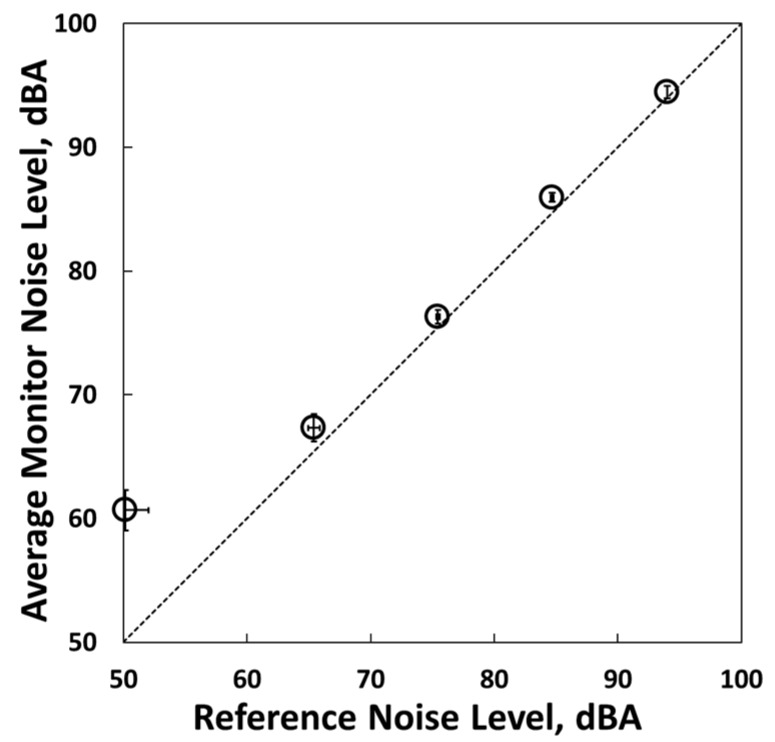
Average noise sensor measurements relative to the reference measurements. The *x*-axis and *y*-axis error bars represent one standard deviation.

A scatterplot of the average concentration levels relative to the sound level meter is shown in Figure 8. The low-cost noise sensor measurements were on the one-to-one line compared to the reference instrument for 60 dBA and above. The 30 sensors were averaged and the *y*-axis error bars represent the standard deviation between sensors. The average slope, intercept, bias, and *R*^2^ for values above 60 dBA were 1.0, −2 dBA, 1.2% and 1.0, respectively, and the precision for all monitors was 1%.

The noise sensor results were strongly correlated with the reference instrument for sound levels between 65 and 95 dBA. The maximum sound level of the correlation test was limited by the experimental apparatus available, but we expect that the sensors are reliable up to 114 dBA, which is at the upper limit of the range recommended by OSHA [44], because the hardware and software should be reliable through that range.

### 3.5. Field Deployment and Samples of Preliminary Data

The factory under study contained a number of manufacturing processes, including arc welding, milling, and painting. The work is generally divided into regions with similar manufacturing processes. The sensors were deployed in the factory in several stages, starting with five monitors and ultimately leading to 40 monitors. Significant effort was invested in making the wireless connectivity of the monitors robust and compatible with the network of the factory in which the devices were deployed. Periodic failures from unique circumstances also led us to develop several layers of fail-safes to restart devices should they fail to consistently record data, ranging from simply retrying the wireless connection to shutting down and restarting the wireless subsystem. These fail-safes themselves occasionally caused bugs but were ultimately helpful in producing a system that could run reliably for months on end, despite the vagaries of the shop environment, including power or internet connection outages, and the constant onslaught of dust, vibration, and oil. A 30-day laboratory test yielded no communication or hardware failures. Over a period of 235 days in the factory, defining a failure as one hour without report, the devices ran with a 10.5 day mean time to failure. Failures were typically repaired by cycling the power. While running, monitors provided an average of 275.8 readings per day, dropping less than 5% of all reports. Dropped reports were stored to the monitor’s flash drive. Because of our limited access to the facility and the facility’s network, it was not possible to further diagnose the source of the system failures.

**Figure 9:**
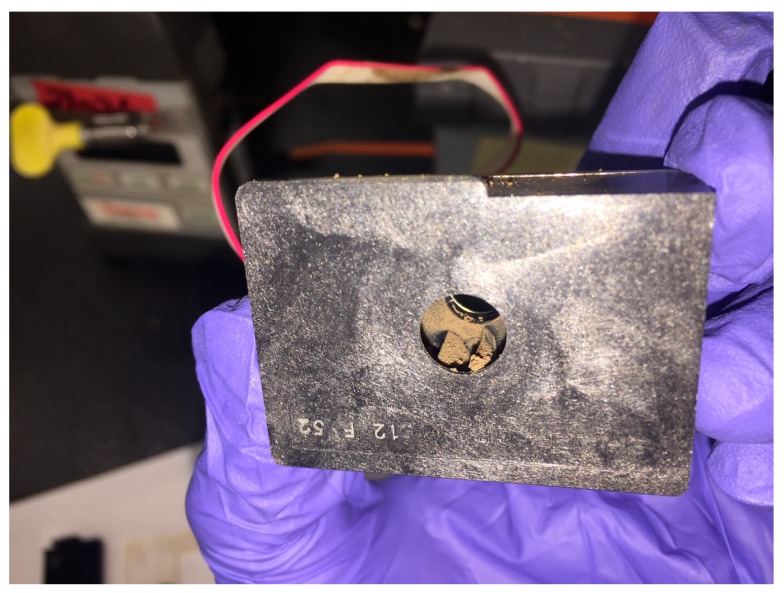
Dust fouled the active Sharp sensor after just 19 weeks of deployment in the factory.

**Figure 10:**
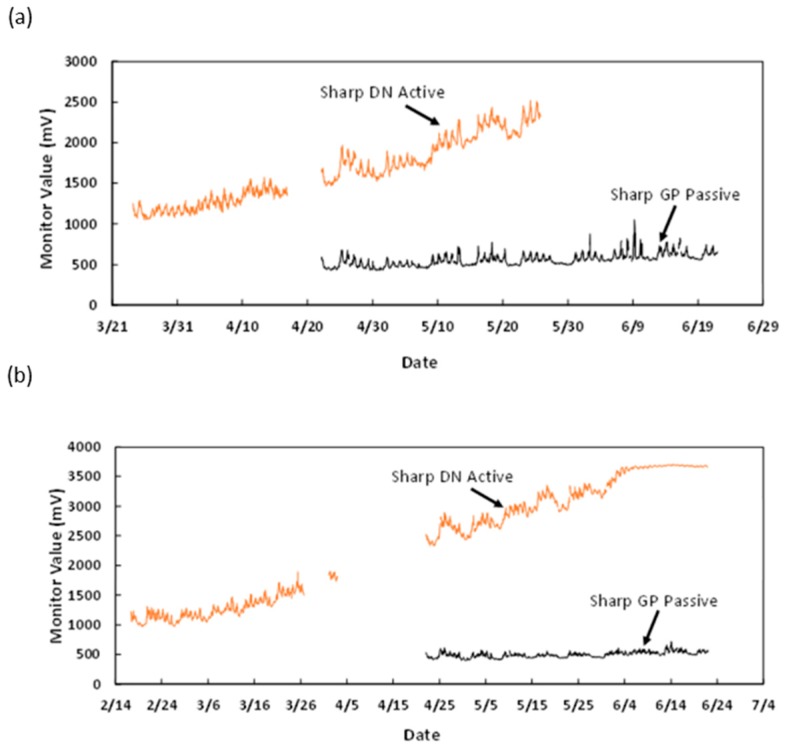
The accumulation of dust on the active Sharp sensors caused the readings to drift upward over time. (**a**) One example pair of collocated active and passive sensors; and (**b**) a second pair of collocated active and passive sensors in which the active sensor signal eventually flatlines. The passive version of the sensors seemed to suffer much less drift. Gaps in the graph represent periods when data were not recorded from the devices.

Somewhat to our surprise, the dust itself became a challenge for the monitors. Figure 9 shows what the interior of the particle sensor looked like after just 19 weeks of deployment in the factory. The readings from this sensor trended steadily upward during deployment, suggesting that the sensor itself was obstructed. We hypothesized that a passive sensor, e.g., one without a fan to pull the air through the sensor, would avoid this heavy contamination. New monitors with passive sensors were constructed and hung in close proximity to the active sensors for several weeks to compare performance. Figure 10 suggests that the passive sensors were less affected by the dust, but a more thorough analysis is underway.

**Figure 11:**
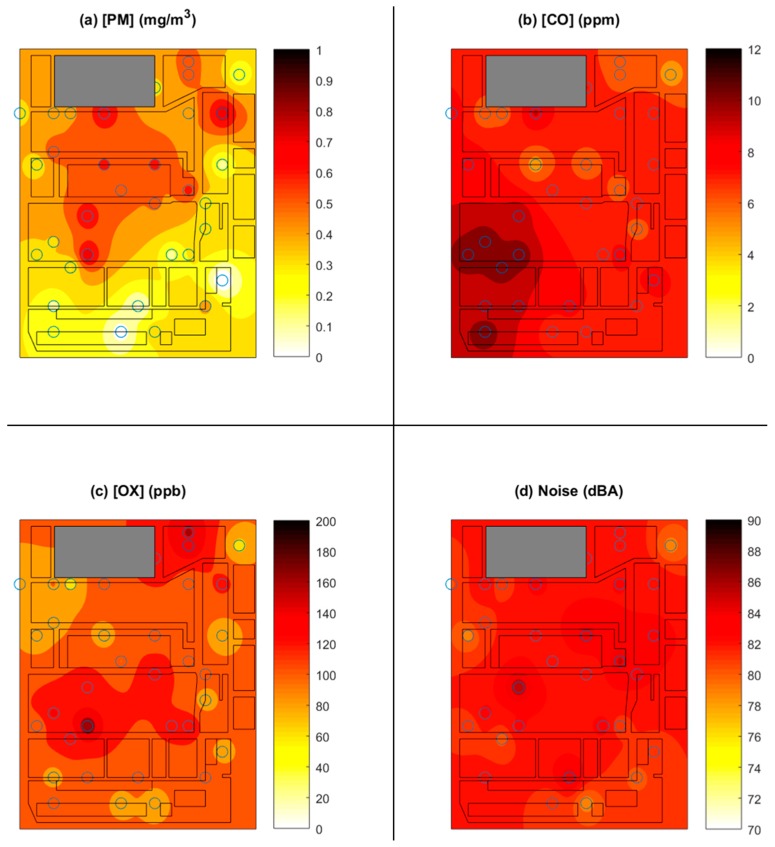
Hazard maps showing the average of (**a**) PM, (**b**) CO, (**c**) oxidizing gases, and (**d**) noise between 8:00 a.m. and 8:55 a.m. on a typical production day. Circles represent the location of the monitors in the factory. An inverse distance weighting scheme was used to estimate hazard levels between monitor locations. The lines indicate work areas in the factory that are generally divided by aisles. The gray-shaded area indicates the office area which is isolated from the manufacturing floor and was not part of the study area. The hue indicates the intensity of the hazard level. The study area is approximately 290 m × 250 m (75,000 m^2^).

**Figure 12:**
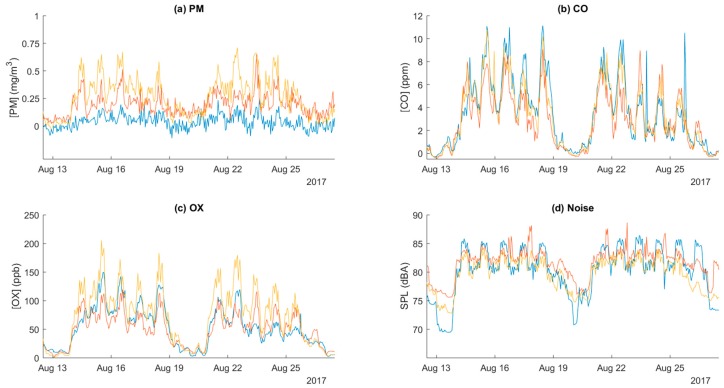
Timeseries plots of (**a**) PM, (**b**) CO, (**c**) oxidizing gases, and (**d**) noise recorded by three monitors at different locations over several days. The two larger patterns indicate work weeks, with hazards being lowest during the weekends. Small peaks within the work week indicate diurnal variation. Differences between the colored lines in a single plot indicate differences caused by the location of the monitors within the factory.

Figure 11 and Figure 12 present samples of the data collected by the network. Figure 11 is an example of four hazard maps constructed from the readings of all of the sensors, averaged over a period of one hour and interpolated between the various sensor locations. Figure 11a displays the variations for the particle sensors, Figure 11b the carbon monoxide sensors, Figure 11c the oxidizing gas sensors, and Figure 11d the noise sensors. This figure makes it clear that the central region, where many welding stations are located, generates the most particles, while the lower left region, where torch cutting equipment is located, was the greatest source of carbon monoxide. Figure 12 illustrates the temporal variations among the four sensor types in three monitors hung at different locations in the factory. Here, the weekly variation and the diurnal variation is unmistakable. The variation among locations can also be appreciated by comparing the three lines within each subfigure.

Other researchers have recognized the advantages of inexpensive sensors that might be used in distributed networks. In 2012, Budde, Busee and Beigl [26] described how a network of dust sensors would be useful for mapping hazards in urban areas and for monitoring long-term personal exposure. In 2013, Javanović et al. [27] went further by developing a prototype monitor based on an inexpensive dust sensor produced by Sharp in 2006 (Sharp, Model GP2Y1010AU0F, Osaka, Japan) [28]. They connected the sensor to a microcontroller and transmitted the signals through an RF transceiver. In 2014, Zakaria et al. [29] also connected the Sharp sensor to a microprocessor, but used the wireless Zigbee communication protocol rather than the ST-TR1100 wireless transceiver used by Javanović et al. Khadem and Sgârciu [30] pursued a similar approach, but connected two different dust sensors: the same Sharp sensor used by Javanović et al., and one manufactured by the Shinyei corporation (Shinyei Corporation, Model PPD4NS, New York, NY, USA), connecting them through an Arduino microcontroller and a wireless transceiver. They measured the responses of both receivers against a third inexpensive commercial device, the Dylos DC1100 (Dylos, Model DC1100, Riverside, CA, USA). Also in 2014, Qian and Cai [31] went still further, integrating a camera, a temperature and humidity sensor, the familiar Sharp sensor, and an air odor sensor (Shenzhen Dovelet Sensors Technology Co., Model TPM-300E, Shenzhen, China). Their prototype-integrated system also communicated wirelessly. A similar prototype developed by Yang et al. in 2015 [32] was based on the Yun microprocessor system and integrated a sensor for volatile organic compounds (Winsen Electronics, Model MQ138, Zhengzhou, China), the aforementioned Sharp dust sensor, and a temperature and humidity sensor (Aosong Electronics, Model AMT2001, Guangzhou, China). The Yun microprocessor acted as a web server, which could then be accessed by another device, such as a cell phone. The monitors described in this work include a suite of instruments specifically selected to monitor aerosol, gas, and noise hazards in a manufacturing environment. The monitors incorporate several new sensors that have not been reported in previous work. Also, this report describes how the system was calibrated in the lab and deployed in the field, which has not been described in previous work.

## 4. Conclusions

The particle sensor, carbon monoxide sensor, oxidizing gas sensor, and noise sensor all performed with accuracy and precision levels consistent with monitoring for multiple hazards in an active factory environment. These were integrated into a plastic case, along with a temperature and humidity sensor, which allowed them to be safely deployed for sustained periods in a sensor network. The data from these sensors were consistently collected and saved to a database.

The network of low-cost monitors demonstrated here increases the data available upon which to base effective risk mitigation strategies. Conventional sampling provides a single measurement of one hazard, typically integrated over a work shift, and only becomes available weeks after sampling. In contrast, the approach presented here provides continuous measurements of multiple hazards throughout the workplace, and these measurements are immediately available for interpretation and action. Such measurements can be used to identify sources of hazards, formulate strategies for improved control, and evaluate the effectiveness of controls over time. Combined with worker location, these measurements can be used to estimate personal exposures for an entire workforce rather than a single person. This departure from the status quo of conventional sampling will enable a shift to comprehensive exposure assessment with substantially greater certainty that workers are adequately protected from workplace hazards.

The data from the system will form the basis of several future studies, including the development of a real-time mapping system to understand how the levels of various hazards change throughout the day and over longer periods, periods in which the air handling requirements change as weather and environmental loads change alongside factory demand. The system will also be used to understand the individual exposure of a single worker and, once integrated with a position tracking system, can be modeled as the worker moves through the factory. The sensor network itself could be improved by optimizing energy efficiency, congestion control, network connectivity and network coverage (e.g., [33,34,35,36]). Finally, we are interested in how data from the network may be used to change individual exposure to workers, and how such data might be utilized to implement control strategies to reduce harmful concentrations and intensities.
